# High Level of Mid-Regional Proadrenomedullin during ST-Segment Elevation Myocardial Infarction Is an Independent Predictor of Adverse Cardiac Events within 90-Day Follow-Up

**DOI:** 10.3390/medicina58070861

**Published:** 2022-06-28

**Authors:** Anggoro Budi Hartopo, Ira Puspitawati, Vita Yanti Anggraeni

**Affiliations:** 1 Department of Cardiology and Vascular Medicine, Faculty of Medicine, Public Health and Nursing, Universitas Gadjah Mada-Dr. Sardjito Hospital, Yogyakarta 55281, Indonesia; 2 Department of Clinical Pathology and Laboratory Medicine, Faculty of Medicine, Public Health and Nursing, Universitas Gadjah Mada-Dr. Sardjito Hospital, Yogyakarta 55281, Indonesia; ipuspitawati@ugm.ac.id; 3 Division of Cardiology, Department of Internal Medicine, Faculty of Medicine, Public Health and Nursing, Universitas Gadjah Mada-Dr. Sardjito Hospital, Yogyakarta 55281, Indonesia; vitayanti@ugm.ac.id

**Keywords:** MR-proADM, ST-elevation myocardial infarction, prognostic, adverse cardiac events

## Abstract

*Background and Objectives*: the cardiovascular adverse events including mortality and heart failure, persist significantly during the first months after the acute phase of ST-segment elevation myocardial infarction (STEMI). The increased level of midregional proadrenomedullin (MR-proADM), at hospital presentation in STEMI patients is considered an independent predictor of short-term and long-term mortality and heart failure. This study aimed to measure MR-proADM levels during the acute and recovery phases of STEMI and corroborate whether MR-proADM level was associated with the adverse cardiac events after recovering from STEMI. *Materials and Methods*: this prospective study enrolled subjects with acute phase STEMI admitted to the intensive cardiac care unit. After recovering and discharged from hospitalization, subjects were followed-up for 90 days. For MR-proADM measurement, the blood samples during acute phase were withdrawn on hospital admission (MR-proADM-0) and during recovery at the day-30 follow up (MR-proADM-30). Adverse cardiac events were evaluated at 30-day and 90-day follow up, namely a composite of death, chronic heart failure, and hospital readmission of any cardiac causes. *Results*: 83 subjects were enrolled. The median MR-proADM-0 was 3313.33 pg/mL and MR-proADM-30 was significantly reduced at 292.50 pg/mL, *p* < 0.001. Nineteen subjects (22.9%) experienced adverse cardiac events at 30-day follow up. The MR-proADM-0 level was independently associated with 30-day adverse cardiac events (adjustedOR 1.002, 95%CI: 1.001–1.003, *p* = 0.040), after adjustment with other variables. In this case, 25 subjects (32.5%) experienced adverse cardiac events at 90-day follow-up. The MR-proADM-0 level was independently associated with 90-day adverse cardiac events (adjustedOR 1.002, 95%CI: 1.001–1.003, *p* = 0.049). The higher changes of MR-proADM-0 to MR-proADM-30 also associated with adverse cardiac events at 90 days. *Conclusions:* The MR-proADM was significantly increased during the acute phase of STEMI and declined during recovery phase. The higher MR-proADM level during the acute phase of STEMI and its change intensity were predictors of adverse cardiac events within the 90-day follow up.

## 1. Introduction

Currently, the outcomes of patients with ST-segment elevation myocardial infarction (STEMI) have improved due to the enhancement of revascularization procedures and prevention strategies starting during acute care [1]. However, the cardiovascular adverse events including mortality and heart failure, persist significantly during the first months after the acute phase of STEMI [2].

Adrenomedullin (ADM) is a 52-amino-acid peptide initially isolated from human pheochromocytoma [3]. It was also discovered in the adrenal medulla, heart, lungs, kidneys, gastrointestinal tract, brain, endothelium, and vascular smooth muscle cells [4,5]. In the cardiovascular system, ADM has a vasodilatory property, augments cardiac output, and stimulates diuresis and natriuresis [5]. Multiple conditions such as shear stress, increased cardiac workload, ischemia, hypoxia, acidosis, catecholamines, angiotensin II, vasopressin, and inflammatory cytokines provoke the secretion of ADM [5]

Adrenomedullin (ADM) is derived from a precursor, proADM [5]. This larger peptide is cleaved to two biologically active peptides, ADM and a 20-aminoacid peptide proadrenomedullin N-terminal 20 peptide (PAMP) [6]. The residual portion is midregional proadrenomedullin (MR-proADM) which is lined by the PAMP and ADM sequences [6]. This stable peptide is secreted in equimolar quantities as ADM and a commonly measured biomarker in this ADM system [7]. The increased level of MR-proADM at hospital presentation in STEMI patients is considered an independent predictor of short-term and long-term mortality and heart failure [8]. There was a solid prognostic value of high MR-proADM level for mortality and non-fatal cardiovascular events in patient with acute myocardial infarction [9,10].

This study aimed to measure MR-proADM levels during the acute and recovery phases of STEMI and corroborate whether MR-proADM level was associated with the adverse cardiac events after recovering from STEMI.

## 2. Materials and Methods

### 2.1. Subjects

This prospective study enrolled subjects who were patients with acute phase STEMI admitted to the intensive cardiac care unit of Dr. Sardjito Hospital, Yogyakarta, Indonesia. The subjects were enrolled consecutively, from January 2017–January 2019. The blood samples were stored in the Biobank Faculty of Medicine, Public Health and Nursing Universitas Gadjah Mada, Yogyakarta, Indonesia. The diagnosis of STEMI was based on the criteria as previously described [11]. The subject’s inclusion criteria were: [1] diagnosis of STEMI, [2] age from 35 to 70 years old, [3] symptom onset ≤ 24 h, and [4] no revascularization performed pre-hospital admission. The subjects were excluded if: [1] had chronic heart failure (NYHA functional class ≥ II), [2] had chronic kidney disease (stages > III), [3] had hepatic cirrhosis, [4] diagnosed with chronic inflammatory diseases (chronic rheumatic disease, and inflammatory bowel disease), [5] had malignancy, [6] had severe acute infection and sepsis during hospitalization, and [7] had acute stroke during hospitalization. All subjects gave informed consent to participate in this study. The protocol of this research had been approved by Medical and Health Research Ethic Committee Faculty of Medicine, Public Health and Nursing, Universitas Gadjah Mada, Yogyakarta, Indonesia (No: KE/0645/05/2019, approval date: 29 May 2019).

### 2.2. Study Protocol

During in-hospital intensive care, the initial and subsequent management were at the discretion of the attending cardiologists based on the hospital standard-of-care. After recovering from acute phase of STEMI and discharged from hospitalization, subjects were followed-up for 90 days. Subjects were asked to visit the study team in our hospital at day 30 and at day 90 after discharge in order to be evaluated regarding the adverse cardiac events and the management during follow-up, by anamnesis. At day 30, the blood samples were withdrawn for biomarker, MR-proADM, measurement. The adverse cardiac events were a composite of death, diagnosis of chronic heart failure, i.e., the persistence of heart failure after acute STEMI, and hospital readmission of any cardiac causes including urgent percutaneous coronary intervention (PCI). Subjects who failed to visit in the 30-day or 90-day follow-up were phone-called or text-messaged to obtain the required data for study follow-up. The study team evaluated the subjects were blinded to the biomarker measurement results.

The data regarding demography, clinical presentation, laboratory results, and treatments during hospitalization were documented in the case report form [11]. The blood samples were taken from the antecubital veins in a supine position on hospital admission before any revascularization treatments (PCI or fibrinolysis). The hematology and blood chemistry examinations were performed in the hospital laboratory as routine procedure. For MR-proADM measurement, the blood samples during acute phase were withdrawn on hospital admission (MR-proADM-0) and during recovery at the day-30 follow-up (MR-proADM-30) which were decanted into vacutainer tubes, left alone at room temperature to clot at 20–30 min, centrifuged for 20 min, and stored at −80 °C in our Biobank until assayed by Human MR-ProADM enzyme linked immunosorbent assay (ELISA) Kit (Elabscience^®^, Houston, TX, USA). The sandwich ELISA procedure was performed by experience technicians in Integrated Research Laboratory Faculty of Medicine, Public Health and Nursing Universitas Gadjah Mada to quantify MR-pro ADM level based on the manufacturer’s instructions.

### 2.3. Statistical Analysis

Subjects were divided into two groups based on adverse cardiac event occurrences at 30-days and 90-days follow-up. The comparison of continuous data between groups was analyzed with Student T-tests, for normally distributed data, and Mann-Whitney tests, for not normally distributed data. Normality analysis was conducted with Kolmogorov-Smirnov test, after log-10 transformation. The difference of categorical data between groups was analyzed with chi-square test or Fisher-exact test, where applicable using means with standard deviation (SD). The univariate and multivariable analyses, by logistic regression method, were performed to associate the MR-proADM and other variables with adverse events at 30-days and 90-days follow-up. The comparison between two related samples was conducted with the Wilcoxon signed rank test, for not normally distributed data. The correlation measurement between continuous variables was performed with Pearson’s correlation test. A *p* value < 0.05 was set as statistically significant.

## 3. Results

### 3.1. MR-proADM Level Changes from Acute to Recovery Phase

There were 83 subjects who were enrolled for this study. Table 1 showed the baseline characteristics of subjects enrolled in this study.

The median acute phase MR-proADM level (MR-proADM-0) was 3313.33 pg/mL on hospital admission. During recovery phase at 30-days after discharge, the median MR-proADM level (MR-proADM-30) was significantly reduced at level 292.50 pg/mL, *p* < 0.001. The mean changes from MR-proADM-0 to MR-proADM-30 was 4509.53 pg/mL. The mean ratio of MR-proADM-0:MR-proADM-30 was 18.7 (as shown in Table 2).

### 3.2. MR-proADM Level and 30-Day Follow Up Adverse Cardiac Events

Among the 83 subjects, 19 subjects (22.9%) experienced adverse cardiac events within recovery 30-day follow up after discharge. The adverse cardiac events consisted of 4 death subjects, 8 chronic heart failure and 7 hospital readmissions due to acute coronary syndrome (ACS) (both STEMI and nonSTEACS). Subjects who experienced 30-day adverse cardiac events had significantly higher MR-proADM-0 level as compared to those without 30-day adverse cardiac events (mean ± SD: 8072.4 ± 7599.5 pg/mL vs. 4188.2 ± 3953.2 pg/mL, *p* = 0.022). The level of MR-proADM-30 did not significantly differ between subjects who experienced 30-day adverse cardiac events and those without (mean ± SD: 442.5 ± 365.0 pg/mL vs. 359.9 ± 262.0 pg/mL, *p* = 171). The changes and ratio of MR-proADM-0 and MR-proADM-30 tended to be higher in subjects who experienced 30-day adverse cardiac events (Table 3).

The MR-proADM-0 level was independently associated with 30-day adverse cardiac events (adjustedOR 1.002, 95%CI: 1.001–1.003, *p* = 0.040), after adjustment with pulse rate (adjustedOR 1.010, 95%CI: 0.972–1.049, *p* = 0.624), urea nitrogen level (adjustedOR 1.047, 95%CI: 0.924–1.186, *p* = 0.474), creatinine level (adjustedOR 1.459, 95%CI: 0.238–8.943, *p* = 0.683) and Killip class (adjustedOR 4.777; 95%CI: 1.005–22.692, *p* = 0.049) (Table 4).

### 3.3. MR-proADM Level and 90-Day Follow Up Adverse Cardiac Events

At the 90-day follow-up, there were 6 subjects who did not visit the study team and did not respond to phone-called or text-messaged. Therefore, 77 subjects were able to be followed-up at 90 days after discharge. Among them, 25 subjects (32.5%) experienced adverse cardiac events. The adverse cardiac events consisted of 6 death subjects, 12 chronic heart failures and 7 hospital readmissions due to acute coronary syndrome (ACS) (both STEMI and non-STEACS). Subjects who experienced 90-day adverse cardiac events had significantly higher MR-proADM-0 level as compared to those without 90-day adverse cardiac events (mean ± SD: 7116.6 ± 6980.2 pg/mL vs. 4116.6 ± 4079.6 pg/mL, *p* = 0.027). The MR-proADM-30 level did not significantly differ between subjects who experienced 90-day adverse cardiac events and those without (mean ± SD: 400.8 ± 325.8 pg/mL vs. 361.1 ± 281.6 pg/mL, *p* = 0.314). The changes of MR-proADM (from MR-proADM-0 to MR-proADM-30) were significantly higher in subjects who experienced 90-day adverse cardiac events. The ratio of MR-proADM tended to be higher in subjects who experienced 90-day adverse cardiac events (Table 5).

The MR-proADM-0 level was independently associated with 90-day adverse cardiac events (adjustedOR 1.002, 95%CI: 1.001–1.003, *p* = 0.049), after adjustment with pulse rate (adjustedOR 1.015, 95%CI: 0.977–1.054, *p* = 0.451), urea nitrogen level (adjustedOR 1.115, 95%CI: 1.013–1.228, *p* = 0.026), creatinine level (adjustedOR 1.564, 95%CI: 0.256–9.553, *p* = 0.628) and hypertension (adjustedOR 5.746; 95%CI: 1.715–19.254, *p* = 0.005) (Table 6). The changes of MR-proADM were not included in the multivariable analysis because of reduced sample size of subjects.

## 4. Discussion

The study found that MR-proADM level was significantly increased during the acute phase of STEMI and declined during recovery phase at day 30 after the STEMI episode. The increased MR-proADM level during acute phase of STEMI was an independent predictor of adverse cardiac events for both at 30-day and at 90-day follow-up after the acute phase of STEMI. The intensity of increased MR-proADM during acute phase of STEMI, reflected by the higher changes of MR-proADM from acute phase to 30-day follow-up, was significantly associated with adverse cardiac events at 90-day follow-up.

As a potent vasodilator and a marker of hemodynamic stress, increased level of MR-proADM in the blood circulation of STEMI patients was associated with worsened prognosis, both in short term at 30 days and long term at 1500 days [8]. This study showed that repeated measurements with 6-h lapses yielded increasing MR-proADM in the acute phase of STEMI [8]. Higher MR-proADM level during acute phase of STEMI was an independent predictor of adverse left ventricular remodeling among PCI-treated STEMI patients [12]. Furthermore, higher MR-proADM was an adverse predictor for improvement of left ventricular function or reverse remodeling after STEMI [12]. Our study corroborated these previous studies and showed that MR-proADM increased in the acute phase of STEMI and steeply decreased in the recovery phase after 30 days.

In the heart, ADM is expressed by cardiomyocytes, cardiac fibroblasts, and endothelia [13]. The ADM expression is exaggerated in cardiomyocytes exposed to simulated ischemia with the aim to reduce cardiomyocyte apoptosis by its paracrine effect [14]. In experimental study of myocardial infarction in mice models, the gene expression and protein level of proADM and ADM were augmented [15]. The proADM and ADM increased expressions in infarcted cardiac tissue led to increased plasma MR-proADM levels in circulation following acute myocardial infarction [15]. ProADM encourages cardiac inflammation but weakens established inflammation during acute myocardial infarction, while ADM has restricted impact on inflammation [15]. Both peptides improve cardiomyocyte survival under ischemia [15]. Another study showed that ADM works at cardiac lymph angiogenesis through reparative properties after acute myocardial injury [16]. Therefore, during the acute phase of STEMI, the upregulation of the ADM system and increased circulation of MR-proADM reflect the intensity of acute stress condition, cardiac inflammation and vascular responses of the ADM system which determine the future cardiac remodeling among STEMI patients.

In STEMI, the revascularization attempts by PCI or fibrinolysis successfully reduced the in-hospital adverse cardiac events and mortality. However, several complications still occur due to hemodynamic disturbances. Previous study showed that higher MR-proADM was an independent predictor of cardiogenic shock, both in early and late phase, among patients with STEMI undergoing revascularization [17]. The presence of cardiogenic shock during the acute phase STEMI increased the in-hospital mortality rate and within 30 days after acute phase [2]. The vasodilation properties of biologically active ADM are well-characterized, in which excess circulatory ADM was associated with depressed vascular function due to excessive vasodilation [18]. In sepsis and septic shock, the ADM and MR-proADM levels increased significantly and were associated with worsened outcomes [19,20]. Our study did not detect the impact of MR-proADM level on hemodynamic disturbance during acute phase of STEMI, due to the inadequate sample size.

The limitations of this study include: [1] the subject sample size was small, [2] the follow-up time was limited until 90 days after the acute phase of STEMI, and [3] the number of subjects lost to follow-up until 90 days remained high (7.2%), on whom we did not perform an analysis. Further study with a larger subject sample size and involving longer follow-up time will be necessary to validate and corroborate the finding of our study.

## 5. Conclusions

In conclusion, the level of MR-proADM was significantly increased during the acute phase of STEMI and declined during recovery phase. The higher MR-proADM level during the acute phase of STEMI and its change intensity was an independent predictor of adverse cardiac events within the 90-day follow-up after the acute phase of STEMI. This study corroborates previous findings of the role of MR-proADM in the prognostication of patients presenting with STEMI.

## Figures and Tables

**Table 1:** The baseline characteristics of subjects.

Parameters	Values (*n* = 83)
Demography and risk factors	
Age (years), mean ± SD	54.6 ± 8.8
Male sex, *n* (%)	79 (95.2)
Body weight (kg), mean ± SD	66.2 ± 13.3
Body mass index, mean ± SD	24.7 ± 4.1
Hypertension, *n* (%)	37 (44.6)
Dyslipidemia, *n* (%)	20 (24.1)
Diabetes mellitus, *n* (%)	18 (21.7)
Coronary heart disease, *n* (%)	16 (19.3)
Current smoker, *n* (%)	56 (67.5)
Ex smoker, *n* (%)	9 (10.8)
ICCU hospitalization, *n* (%)	4 (4.8)
Obesity, *n* (%)	33 (39.8)
Clinical pictures	
Onset (hours), mean ± SD	5.8 ± 4.2
Systolic blood pressure,	133.9 ± 25.3
Diastolic blood pressure,	83.2 ± 13.7
Pulse rate,	79.7 ± 18.0
Killip class II, *n* (%)	9 (10.8)
Killip class III, *n* (%)	5 (6.0)
Anterior infarction, *n* (%)	50 (60.2)
Laboratory examination	
Hemoglobin (g/dL),	14.5 ± 1.5
Leucocytes (counts/mm^3^)	13.9 ± 3.6
Platelet (counts/mm^3^)	267.4 ± 84.9
Urea nitrogen (g/dL)	15.3 ± 6.3
Creatinine (mg/dL)	1.3 ± 0.5
Glucose (g/dL)	168.1 ± 75.2
HbA1c (%)	6.7 ± 1.8
Medications	
Primary PCI	56 (67.5)
Successful fibrinolysis	21 (25.3)
Heparin	54 (65.1)
ACE-inhibitor	56 (67.5)
Beta blocker	48 (57.8)

SD: standard deviation; ICCU: intensive cardiac care unit; HbA1c: glycated hemoglobin; PCI: percutaneous coronary intervention; ACE: angiotensin-converting enzyme.

**Table 2:** The MR-proADM levels on hospital admission and at 30-day follow-up.

MR-proADM Level, pg/mL *	On Hospital Admission, MR-proADM-0 (*n* = 83)	At 30-Day Follow-Up, MR-proADM-30 (*n* = 72)
Mean	5077.37	374.79
Standard deviation	5232.59	282.20
Median	3313.33	292.50
Minimum	242.50	117.50
Maximum	28,202.22	1517.50
Changes (Δ) (mean)	N.A.	4509.53
Ratio (mean)	N.A.	18.7

* The Wilcoxon signed rank test for comparison between MR-proADM value on hospital admission (MR-proADM-0) vs. at 30-day follow up (MR-proADM-30) yielded *p* value < 0.001 (for *n* = 72 subjects each). MR-proADM: mid regional pro adrenomedullin; N.A.: not applicable.

**Table 3:** The characteristics of subjects divided by the presence and the absence of 30-day follow up adverse cardiac events.

Parameters	30-Day Adverse Cardiac Event (*n* = 19)	30-Day No Adverse Cardiac Event (*n* = 64)	*p* Value
Demography and risk factors			
Age (years), mean ± SD	54.8 ± 8.8	54.6 ± 8.8	0.458
Male sex, *n* (%)	18 (94.7)	61 (95.3)	0.654
Body weight (kg), mean ± SD	66.4 ± 14.5	66.1 ± 13.1	0.466
Body mass index, mean ± SD	25.5 ± 4.4	24.4 ± 3.9	0.322
Hypertension, *n* (%)	12 (63.2)	25 (39.1)	0.064
Dyslipidemia, *n* (%)	3 (15.8)	17 (26.6)	0.261
Diabetes mellitus, *n* (%)	4 (21.1)	14 (21.9)	0.607
Coronary heart disease, *n* (%)	3 (15.8)	13 (20.3)	0.473
Current smoker, *n* (%)	14 (73.7)	42 (65.6)	0.653
Ex smoker, *n* (%)	1 (5.3)	8 (12.5)	0.653
ICCU hospitalization, *n* (%)	1 (5.3)	3 (4.7)	0.654
Obesity, *n* (%)	10 (52.6)	23 (35.9)	0.192
Clinical pictures			
Onset (hours), mean ± SD	6.5 ± 5.3	5.6 ± 3.8	0.441
Systolic blood pressure,	132.3 ± 22.7	134.4 ± 26.2	0.372
Diastolic blood pressure,	86.9 ± 12.5	82.1 ± 13.9	0.093
Pulse rate,	87.5 ± 15.3	77.3 ± 18.2	0.015
Killip class II, *n* (%)	4 (21.1)	5 (7.8)	0.024
Killip class III, *n* (%)	3 (15.8)	2 (3.1)	0.024
Anterior infarction, *n* (%)	10 (52.6)	40 (62.5)	0.158
Laboratory examination			
Hemoglobin (g/dL),	14.3 ± 1.3	14.6 ± 1.6	0.299
Leucocytes (counts/mm^3^)	13.9 ± 3.4	13.9 ± 3.7	0.448
Platelet (counts/mm^3^)	249.1 ± 72.4	272.8 ± 88.1	0.143
Urea nitrogen (g/dL)	18.1 ± 8.4	14.5 ± 5.3	0.044
Creatinine (mg/dL)	1.5 ± 0.6	1.2 ± 0.4	0.016
Glucose (g/dL)	169.1 ± 82.9	167.9 ± 73.6	0.476
HbA1c (%)	6.8 ± 2.2	6.5 ± 1.7	0.316
Medications			
Primary PCI	12 (63.2)	44 (68.8)	0.648
Successful fibrinolysis	4 (21.1)	17 (26.6)	0.194
Heparin	14 (73.7)	40 (62.5)	0.369
ACE-inhibitor	14 (73.7)	42 (65.6)	0.510
Beta blocker	10 (52.6)	38 (59.4)	0.601
Biomarkers			
MR-proADM-0	8072.4 ± 7599.5	4188.2 ± 3953.2	0.022
MR-proADM-30 *	442.5 ± 365.0	359.9 ± 262.0	0.171
Changes (Δ) MR-proADM *	7077.7 ± 6370.4	3943.7 ± 4109.1	0.056
Ratio MR-proADM *	22.3 ± 17.2	17.9 ± 25.4	0.279

* Subjects with 30-day events (*n* = 13), no events (*n* = 59). SD: standard deviation; ICCU: intensive cardiac care unit; HbA1c: glycated hemoglobin; PCI: percutaneous coronary intervention; ACE: angiotensin-converting enzyme; MR-proADM: mid-regional pro adrenomedullin; MR-proADM-0: mid-regional pro adrenomedullin on admission; MR-proADM-30: mid-regional pro adrenomedullin at day 30 after discharge.

**Table 4:** Univariate and multivariable analysis for 30-day follow up adverse cardiac events.

Covariables	Unadjusted OR	95% (CI)	*p* Value	Adjusted OR	95% (CI)	*p* Value
MR-proADM-0	1.001	1.000–1.002	0.013	1.002	1.001–1.003	0.040
Pulse rate	1.036	1.002–1.071	0.035	1.010	0.972–1.049	0.624
Urea nitrogen	1.087	1.006–1.176	0.035	1.047	0.924–1.186	0.474
Creatinine	4.100	1.380–12.180	0.011	1.459	0.238–8.943	0.683
Killip class ≥ 2	4.750	1.404–16.067	0.012	4.777	1.005–22.692	0.049

OR: odd ratio; CI: confidence interval; MR-proADM-0: mid-regional pro adrenomedullin on admission.

**Table 5:** The characteristics of subjects divided by the presence and the absence of 90-day follow up adverse cardiac events.

Parameters	90-Day Adverse Cardiac Event (*n* = 25)	90-Day No Adverse Cardiac Event (*n* = 52)	*p* Value
Demography and risk factors			
Age (years), mean ± SD	55.5 ± 8.3	54.9 ± 8.5	0.376
Male sex, *n* (%)	23 (92.0)	50 (96.2)	0.442
Body weight (kg), mean ± SD	65.1 ± 13.8	66.9 ± 13.2	0.295
Body mass index, mean ± SD	24.7 ± 4.4	24.7 ± 4.1	0.322
Hypertension, *n* (%)	17 (68.0)	19 (36.5)	0.010
Dyslipidemia, *n* (%)	5 (20.0)	13 (25.0)	0.627
Diabetes mellitus, *n* (%)	6 (24.0)	11 (21.2)	0.778
Coronary heart disease, *n* (%)	4 (16.0)	11 (21.2)	0.762
Current smoker, *n* (%)	17 (68.0)	34 (65.4)	0.893
Ex smoker, *n* (%)	2 (8.0)	6 (11.5)	0.893
ICCU hospitalization, *n* (%)	1 (4.0)	3 (5.8)	0.608
Obesity, *n* (%)	11 (44.0)	21 (40.4)	0.763
Clinical pictures			
Onset (hours), mean ± SD	7.1 ± 5.5	5.4 ± 3.6	0.085
Systolic blood pressure,	131.2 ± 20.7	134.9 ± 24.1	0.254
Diastolic blood pressure,	85.8 ± 11.9	82.9 ± 13.2	0.190
Pulse rate,	88.4 ± 14.7	77.9 ± 18.6	0.009
Killip class II, *n* (%)	4 (16.0)	5 (9.6)	0.109
Killip class III, *n* (%)	3 (12.0)	1 (1.9)	0.109
Anterior infarction, *n* (%)	12 (48.0)	37 (71.2)	0.072
Laboratory examination			
Hemoglobin (g/dL),	14.4 ± 1.4	14.6 ± 1.7	0.343
Leucocytes (count/mm^3^)	13.7 ± 3.1	13.9 ± 3.9	0.400
Platelet (count/mm^3^)	259.9 ± 69.8	270.3 ± 91.8	0.309
Urea nitrogen (g/dL)	17.7 ± 7.6	14.1 ± 5.2	0.019
Creatinine (mg/dL)	1.4 ± 0.5	1.2 ± 0.4	0.017
Glucose (g/dL)	180.8 ± 91.0	169.9 ± 78.0	0.300
HbA1c (%)	7.0 ± 2.3	6.5 ± 1.7	0.185
Medications			
Primary PCI	17 (68.0)	36 (69.2)	0.913
Successful fibrinolysis	4 (16.0)	16 (30.8)	0.152
Heparin	17 (68.0)	31 (59.6)	0.477
ACE-inhibitor	16 (64.0)	37 (71.2)	0.526
Beta blocker	11 (44.0)	34 (65.4)	0.075
Biomarkers			
MR-proADM-0	7116.6 ± 6980.2	4116.6 ± 4079.6	0.027
MR-proADM-30 *	400.8 ± 325.8	361.1 ± 281.6	0.314
Changes (Δ) MR-proADM *	6348.3 ± 5730.3	3892.6 ± 4268.7	0.032
Ratio MR-proADM *	22.5 ± 17.6	17.9 ± 26.9	0.252

* Subjects with 90-day adverse cardiac events (*n* = 18) and no events (*n* = 47). SD: standard deviation; ICCU: intensive cardiac care unit; HbA1c: glycated hemoglo-bin; PCI: percutaneous coronary intervention; ACE: angiotensin-converting enzyme; MR-proADM: mid-regional pro adrenomedullin; MR-proADM-0: mid-regional pro adrenomedullin on admission; MR-proADM-30: mid-regional pro adrenomedullin at day 30 after discharge.

**Table 6:** Univariate and multivariable analysis for 90-day follow up adverse cardiac events.

Covariables	Unadjusted OR	95% (CI)	*p* Value	Adjusted OR	95% (CI)	*p* Value
MR-proADM-0	1.001	1.000–1.002	0.036	1.002	1.001–1.003	0.049
Pulse rate	1.037	1.005–1.070	0.023	1.015	0.977–1.054	0.451
Urea nitrogen	1.098	1.011–1.191	0.026	1.115	1.013–1.228	0.026
Creatinine	3.306	1.153–9.481	0.026	1.564	0.256–9.553	0.628
Hypertension	3.691	1.341–10.157	0.011	5.746	1.715–19.254	0.005

OR: odd ratio; CI: confidence interval; MR-proADM-0: mid-regional pro adrenomedullin on admission.

## Data Availability

The data presented in this study are available on request from the corresponding author. The data are not publicly available due to the confidential nature of data availability.
